# Relationship of Different Perceived Exertion Scales in Walking or Running With Self-Selected and Imposed Intensity

**DOI:** 10.2478/hukin-2014-0100

**Published:** 2014-11-12

**Authors:** Marcelo Ricardo Cabral Dias, Roberto Simão, Geraldo Heleno Ribeiro Machado, Hélio Furtado, Nelson Fortuna Sousa, Helder Miguel Fernandes, Francisco José Félix Saavedra

**Affiliations:** 1 Laboratory of Exercise Physiology and Morphofunctional Assessment, Granbery Methodist College, Juiz de Fora, Brazil.; 2 School of Physical Education and Sports, Rio de Janeiro Federal University, Rio de Janeiro, Brazil.; 3 Post Graduate Program in Sport Science, University of Trás-os-Montes and Alto Douro (UTAD), Vila Real, Portugal.; 4 Castelo Branco University; Secretariat for Healthy Aging and Quality of Life, City Council Rio de Janeiro, Brazil.; 5 Research Center for Sport, Health, and Human Development, University of Trás-os-Montes and Alto Douro (UTAD), Vila Real, Portugal.

**Keywords:** exercise load, RPE, self-efficacy, self-regulation

## Abstract

The aims of this study were to: (1) compare the Heart Rate (HR) and Rating Perceived Exertion (RPE) in training with self-selected and imposed loads, and (2) associate the OMNI-Walk/Run and Borg scales with self-selected and imposed loads, both on a treadmill. Ten trained men (20.3 ± 2.0 years, 75.6 ± 9.8 kg, 175.1 ± 5.1 cm) participated in a training program with self-selected load (time and speed individually preferred) and another with imposed load (even self-selected time and speed 10% higher). The HR and RPE were measured, every minute of training, by the OMNI-Walk/Run and Borg scales. No significant differences were found in the HR and RPE between training sessions. The correlation between the OMNI-Walk/Run and Borg scales showed a moderate association (r = 0.55) in training with self-selected load and a strong association in imposed load (r = 0.79). In this study, self-selected load induced a suboptimal stimulus to elicit favorable organic adaptations. Moreover, high correlation of OMNI Walk/Run and Borg scales with the imposed load showed that the greater the load of training the best were answers of RPE.

## Introduction

With exercise, the load of training should generate stimuli above that limit which the body is already used to take on the day-to-day basis. Walking and running are traditional exercise types, which promote healthy lifestyle and fitness (ACSM, 2011). There has been an increasing number of people in gyms whose training is comprised of walking and/or running. Therefore, due to lack of monitoring the exercise intensity, they are often unable to reach their training objectives. However, walkers and runners differ in their exercise intensities which, when self-selected, may be related to psychological dimensions, and situational influences (Pintar et al., 2006; Ekkekakis et al., 2008). For individuals to maintain a training routine, it is necessary to establish some basic components such as a correct load and duration of exercise. The majority of practitioners (86%) use some form of perceived exertion to monitor exercise intensity, rather than objectively control the intensity by means of HR monitoring (7%) (Johnson and Phipps, 2006).

From the standpoint of exercise intensity, one of the underlying assumptions is that lower doses of activities are better tolerated for non-athletes, leading to greater involvement and an adherence rate (Ekkekakis, 2009). A smaller initial engagement in physical activity programs and a higher dropout rate have been associated with higher exercise loads (Dishman et al., 1994; Lind et al., 2005). Buzzachera et al. (2007) found that subjects who trained with moderate and high load, differed in intensities previously recommended. In this sense, Vazou-Ekkekakis and Ekkekakis (2009) observed that when the training load was imposed by a coach, there was a difference in the HR and Rating of Perceived Exertion (RPE) in relation to training with a self-selected load. Therefore, different RPE scales are used (Silva et al., 2001; Kilpatrick et al., 2009; Wardwell et al., 2013). Thus, to test the applicability of the prescription of exercise intensity through the RPE scales and the relationship between them for different walking / running intensities becomes necessary.

To measure the RPE, commonly, the Borg scale is widely used for this purpose (Borg, 1982; Johnson and Phipps, 2006), but other scales have been described and recommended with greater specificity (ACSM, 2011). The OMNI scale was developed for various physical exercises, including walking/running – OMNI-Walk/Run (Utter et al., 2004). The construct and concurrent validity analyses have demonstrated that the OMNI-Walk/Run scale has high overall correlation with the Borg scale, pointing as an alternative to control the intensity of treadmill training.

The OMNI-Walk/Run and Borg scales have a higher direct correspondence with the tests of maximum effort and lower with training and a great reference for training of non-athletes with low and high physical capacity. However, Silva et al. (2011) found that there were significant differences in the HR, in exercise performed on a cycle ergometer, for some categories of the OMNI and Borg scales. And yet, few studies have investigated the ratio of preferred intensity, affective and perceptual responses at self-intensity or imposed. No study has analyzed different training loads and RPE with self-selected and imposed intensity in healthy non athletes. In this sense, little has been studied about the relationship of the scales of RPE in walkers or runners with self-selected and imposed loads. Thus, the objectives of this study were to: (1) compare the HR and RPE in training with self-selected and imposed loads, and (2) associate the OMNI-Walk/Run and Borg scales in training with self-selected and imposed loads, both on a treadmill.

## Material and Methods

### Experimental Approach to the Problem

Participants underwent two walking or running training sessions with a self-selected load (time and speed preferably) and one other with an imposed load (even self-selected time and speed 10% higher). The HR and RPE were measured by the OMNI-Walk/Run and Borg scales every minute of training for each condition.

### Subjects

Ten trained men (20.3 ± 2.0 years, 75.6 ± 9.8 kg, 175 1 ± 5.1 cm) were randomly selected from a convenience sample of walkers or runners from two gyms. The subjects performed a walk or a run, with regularity of at least three days a week for 6 months prior to the study. Those who had some muscle or joint limitations that might affect performance during training sessions were excluded from the sample. All participants signed an informed consent form. The study design was prepared in accordance with the Declaration of Helsinki of 1964 as revised in 2008. The procedures were revised and accepted by a local research ethics committee.

### Procedures

The subjects underwent three sessions of walking or running on a treadmill (Movement®, LX160, Brazil) on different days with an interval of 24 to 48 hours. All sessions were held in the morning. In the first session, for familiarization procedures and to the RPE scales, all subjects exercised for 10 min with individual speed preference; every minute the participants were presented the RPE scales (OMNI or Borg) alternately. In the next session, training was performed with a self-selected load, in which subjects chose a time and speed that they normally selected in their practice sessions. In this session, they could adjust the speed at any time. In the third session, the subjects performed training with an imposed load, using the same time, but with an imposed speed, 10% higher than the one in the self-selected session. In this case, they could not adjust the speed of a treadmill, except in a situation of volitional fatigue.

### Heart rate

The HR was measured continuously, through a heart monitor (Polar®, RS400, Finland), every minute, while conducting both training sessions (self-selected and imposed loads) on the treadmill. The maximum HR (HR_max_) was calculated using the equation suggested by Tanaka et al. (2001): 208 - (0.7 × age). The highest HR achieved during training was called peak HR of training (FC_peak.training_), beyond the mean HR of all training (HR_mean_).

### Rating of Perceived Exertion

The RPE was measured by the administration of the OMMI-Walk/Run 0–10 (Utter et al., 2004) and Borg 6–12 scales (Borg, 1982) every minute. Both scales had been previously validated by construct and concurrent validation procedures for walking/running.

The OMNI-Walk/Run scale was used with a response scale of 0 (extremely easy) to 10 points (extremely difficult). This scale has, in addition to numerical indications, pictures of effort, which represent an individual walking or running, whose posture changes as they increase the scale categories, conveying impression of greater effort. The Borg scale measures the level of perceived effort using a single numerical and descriptive indicator of effort, ranging from 6 (no exertion) to 20 points (maximal exertion).

### Statistical Analysis

Initially, the internal consistency by *Intraclass Correlation Coefficient* (ICC) for quantitative variables (HR_peak.training_ and HR_mean_) and the *Chronbach’s Alpha* for RPE scales were tested. To verify the data distribution, the test of *Shapiro-Wilk* was performed, which revealed that the variables had normal distribution. In this case, the mean and standard deviation for all variables were calculated.

Paired *t* tests to verify the differences of variables within and between (HR_max_ and HR_peak.training_) and between training sessions (speed, distance, HR_peak.training_, HR_mean_, and RPE scales) were performed. The relationship between the HR and RPE scales with a self-selected and imposed load was examined using Pearson correlation. The significance was set at *p* < 0.05 using the statistical software SPSS 20.0 for Mac (SPSS, Inc., Chicago, IL).

## Results

The ICC was high (r > 0.90) confirming internal consistency of HRpeak.training (r = 0.93) and HRmean (r = 0.97). The Chronbach’s alpha of 0.63 for the OMNI-Walk/Run scale and 0.64 for Borg scale showed weak consistency (0.6–0.7).

The mean ± standard deviation of speed, distance, HRpeak.training, HRmean, and RPE scales are presented in Table 1. In training with an imposed load, the speed of walking/running and the distance was greater than with self-selected load (p < 0.001). However, no significant differences were observed in HRpeak.training (p = 0.542), HRmean (p = 0.352), OMNI-Walk/Run (p = 0.204), and Borg scales (p = 0.497) between training with a self-selected and imposed load.

The estimated HRmax was 193.8 ± 1.4 bpm. The HRpeak,training represented an average for the 91.7 ± 8.6% and 93.2 ± 9.3% of HRmax in training with a self-selected and imposed load, respectively. The HRmean represented 81.4 ± 8.8% (self-selected) and 82.1 ± 10.0% (imposed) of HRmax. The HRpeak,training (p < 0.001) and HRmean (p < 0.05) showed to be significantly lower than HRmax in both training loads.

**Figure 1:**
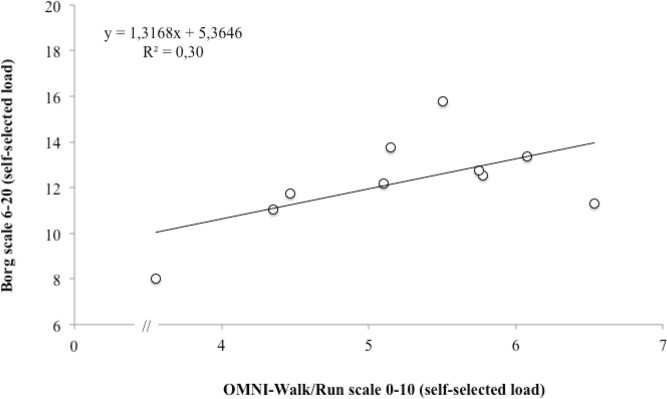
The relationship between the OMNI-Walk/Run and Borg scales in training with a self-selected load.

**Figure 2:**
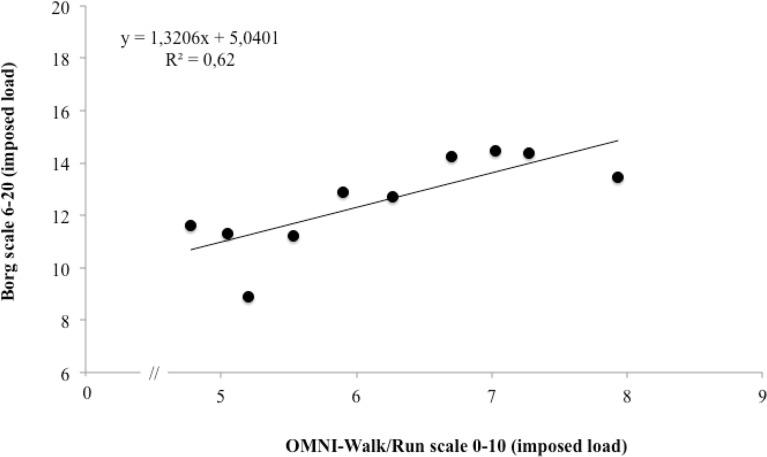
The relationship between the OMNI-Walk/Run and Borg scales in training with an imposed load.

The OMNI-Walk/Run and Borg scales were significantly correlated (p < 0.05) in the imposed load condition (p = 0.007; r = 0.79) when compared to the self-selected load (p < 0.049; r = 0.55). The correlation between the scales is represented by scatter plots (Figures 1 and 2).

**Figure 3:**
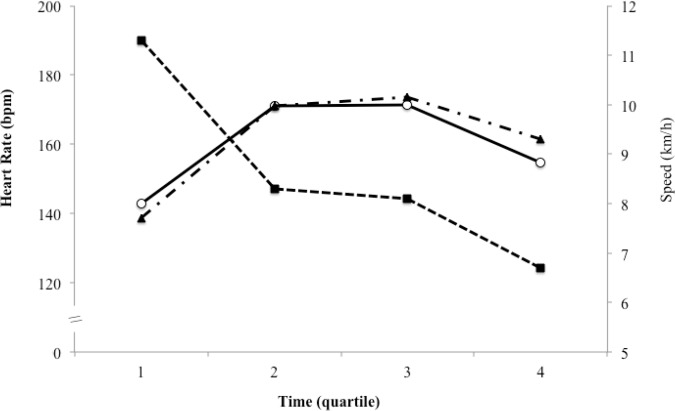
Behavior of Heart Rate relative speed of walking/running with self-selected and imposed loads on the exercise time. 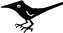 - Speed walking/running; ○ - Training with a self-selected load; ▲ - Training with an imposed load.

**Figure 4:**
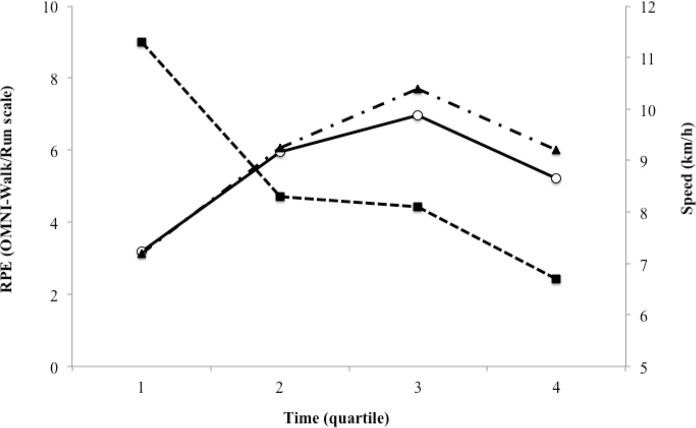
Behavior of OMNI-Walk/Run scale relative speed of walking/running with self-selected and imposed loads on the exercise time. RPE: Rating of Perceived Exertion; 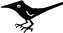 - Speed walking/running; ○ - Training with a self-selected load; ▲ - Training with an imposed load.

**Figure 5:**
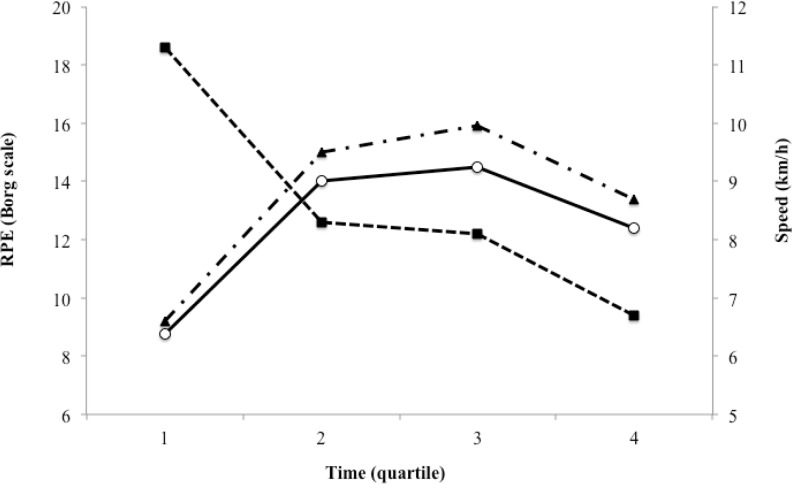
Behavior of Borg scale relative speed of walking/running with self-selected and imposed loads on the exercise time. RPE: Rating of Perceived Exertion; 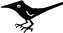 - Speed walking/running; ○ - Training with a self-selected load; ▲ - Training with an imposed load.

The OMNI-Walk/Run and Borg scales were not correlated with the HR during both training procedures (self-selected load: p = 0.183; and imposed load: p = 0.104). Figures 3, 4 and 5 show the relationship of the speed of walking/running with the HR, OMNI-Walk/Run and Borg scales.

## Discussion

The present study indicated that HRpeak,training, HRmean and RPE (OMNI-Walk/Run and Borg) were not different between training with self-selected and imposed loads. Possibly, the speed of walking/running is an important component of this understanding. As the speed of walking/running with imposed load was 10% higher than the self-selected, it became clear that the total distance was significantly greater with the imposed load (Table 1). According to Lind et al. (2008), a load 10% larger than the self-selected one is sufficient to induce significant adaptive responses. On the other hand, in the present study, same with 10% higher than self-selected load, the subjects were able to maintain the activity, without interruption or volitional fatigue, but the HR and RPE were not different between workouts. Thus, the self-selected load seems to remain below what subjects can actually tolerate. For Dishman et al. (1994), subjects should select higher exercise intensities, near their ventilatory threshold, i.e., the point at which there is a sudden change in the patterns of gas exchange (oxygen uptake and carbon dioxide release) during exercise.

Wardwell et al. (2013) found that RPE had a good relationship with self-selected and imposed loads (10% below and above of self-selected), when focusing on measuring intensity. Furthermore, another explanation should be considered. The ICC found that the measurements of HRpeak,training and HRmean during training had high internal consistency, which responded as exercise effort. Likewise, the Chronbach’s Alpha showed that the RPE scales presented weak internal consistency, indicating that responses of the session of training with a self-selected load did not have much consistency with the session with an imposed load. Siegel and Johnson (1992) identified that many subjects tended to minimize or exaggerate their physical symptoms of fatigue through the RPE. In this case, accuracy of RPE may be affected by the exercise intensity. Applying lower loads makes understanding of perceived exertion more difficult. According to Kilpatrick et al. (2009), the RPE scales are also related to the time of exercise, therefore, in the present study, the time also was a self-selected variable.

The HR was not correlated with RPE during training, which in turn showed submaximal intensity characteristics. The ACSM (2011) indicate that, adults should exercise five days a week for 30 to 60 min of moderate aerobic physical activity (64–76% of HRmax or category 12–13 on the Borg scale) or three days of 20 to 60 minutes of high intensity activity (77–95% of HRmax or category 14–17 on the Borg scale), but interpretation of intensity can vary considerably between subjects. According to Dishman et al. (1994), the HR measurements have an error of ± 11 bpm for about 30% of the population. With the possibility of a self-selected load during activity, RPE becomes a critical variable that influences the exercise responses (Dishman et al., 1994).

In the present study, the relationship between the RPE scales was high (r = 0.79), therefore only for training with an imposed load (Figures 1 and 2). During training sessions with self-selected loads, changes in the RPE remain below that of training with imposed loads (Whithers et al., 2006). Training comprised of high intensity activities allows better understanding of the effort and applying the RPE scales is more transparent (Glass and Chvala, 2001; Kilpatrick et al, 2009). According to Silva et al. (2011), it is difficult for individuals to assign exact values to lower categories of the RPE scales. The last category of greater effort intensity, between the two scales, was related. In the latter stages, increases in HR, central and peripheral fatigue, ventilation and overall fatigue enable the subject to identify changes in RPE equally in both scales.

Subjects tolerated training with imposed intensity without achieving volitional fatigue, featuring self-selected intensity as a low stimulus of exercise. Duncan et al. (2005) and Lind et al. (2008) found that training with an imposed load resulted in greater improvements in cardiorespiratory fitness. However, the literature presents several controversial studies about how the self-selected load of walking/running can generate stimuli suitable for the improvement or maintenance of cardiorespiratory fitness (Glass and Chvala, 2001; Pintar et al., 2006; Buzzachera et al., 2007; Ekkekakis, 2009). Some results indicate that individuals who walk or run on a treadmill with a self-selected load maintain the work rates within a moderate range according to the guidelines of the American College of Sports Medicine (Glass and Chvala, 2001; Ekkekakis, 2009). Other studies consider the self selected load beneficial only for subjects with low physical fitness (Pintar et al., 2006; Vazou-Ekkekakis and Ekkekakis, 2009).

When analyzing the speed of walking/running, there was a decrease of 26.5% in speed from the 1st to the 2nd quartile of activity time, then maintenance from the 2nd to the 3rd quartile, and again, a fall of 17.3% from the 3rd to the 4th quartile. These decreases seem to occur because of a stronger start and the difficulty of maintaining the physical effort at given intensity by non-athletes. The HR showed an increase from the 1st to the 2nd quartile and a decrease, only, after the 3rd quartile (Figure 3). It is estimated that even after lowering the speed of the walk/run, the cardiovascular system remains high due to a high stimulus at the beginning of the year (Ekkekakis, 2009).

Although the results of the present study contribute to better understanding of the relationship of different RPE scales with self-selected and imposed training loads (Figure 4 and 5), there are several limitations of the study that should be considered when interpreting the results. The first limitation is associated with the sample, which was small and narrow in terms of representativeness (including only men). Secondly, the experimental design was not balanced between workouts, i.e. since we wanted to monitor the load, we first performed a session with a self-selected load, and then performed a session with an imposed load. Moreover, a lack of measurement of oxygen uptake during training sessions leaves a gap for further discussions about the exercise intensity, considering that this measure would not affect the results and decision-making with regard to the RPE scales.

Taking into consideration that there was no difference in the HR and RPE between training with a self-selected and imposed load, it was concluded that training with a self-selected load owned a suboptimal stimulus to cause favorable adaptations, because the subjects carried out training with imposed load without increasing intensity. Moreover, high correlation of OMNI-Walk/Run and Borg scales with the imposed load showed that the greater the training load, the better were the RPE answers.

## Practical Applications

The results of this study can be of great importance for managers and practitioners with regard to prescription of better training intensity relating to a RPE range. In this case, besides using the HR as a variable to measure the intensity of a workout, the use of the RPE scales during training is encouraged, mainly to control the load level (self-selected or imposed).

## Figures and Tables

**Table 1:** Mean values ± standard deviation of speed, distance, HR_peak.training_, HR_mean_, and RPE scales in training with self-selected and imposed loads

	Self-Selected	Imposed
Speed	7.7 ± 1.7 km/h	8.5 ± 1.9 km/h^*^
Distance	3.6 ± 1.0 km	4.0 ± 1.1 km^*^
HR_peak.training_	177.7 ±17.1 bpm^**^	180.6 ± 18.5 bpm^***^
HR_mean_	157.8 ± 17.4 bpm^#^	159.1 ± 19.7 bpm^#^
RPE (OMNI-Walk/Run 0–10)	5.2 ± 0.9	5.7 ± 1.1
RPE (Borg 6–20)	12.2 ± 2.0	12.5 ± 1.8

HR_peak.training_: Heart Rate peak of training; HR_mean_: Heart Rate media of training; RPE: Rating of Perceived Exertion;

# Significant difference in relation to HR_max_ (p < 0.001);

* Significant difference compared to a self-selected load (p < 0.001);

** Significant difference in relation to HR_max_ (p = 0.013);

*** Significant difference in relation to HR_max_ (p = 0.046).
